# Circadian disruption alters gut barrier integrity via a ß-catenin-MMP-related pathway

**DOI:** 10.1007/s11010-022-04536-8

**Published:** 2022-08-17

**Authors:** Sung Yong Eum, Nicolette Schurhoff, Timea Teglas, Gretchen Wolff, Michal Toborek

**Affiliations:** 1 Department of Biochemistry and Molecular Biology, University of Miami Miller School of Medicine, Miami, FL 33155 USA; 2 Present Address: Institute for Diabetes and Cancer (IDC), Helmholtz Diabetes Center, Helmholtz Centre Munich, Neuherberg, Germany; 3 Present Address: Joint Heidelberg-IDC Translational Diabetes Program, Inner Medicine 1, Heidelberg University Hospital, Heidelberg, Germany; 4 Institute of Physiotherapy and Health Sciences, The Jerzy Kukuczka Academy of Physical Education, 40-065 Katowice, Poland; 5 Department of Biochemistry and Molecular Biology, University of Miami Miller School of Medicine, Suite 528, 1011 NW 15th Street, Miami, FL 33136 USA

**Keywords:** Circadian rhythm disruption, Intestinal barrier integrity, ß-Catenin, Tight junction proteins, Circadian clock genes, Circadian rhythm molecules

## Abstract

We evaluated the mechanistic link between circadian rhythms and gut barrier permeability. Mice were subjected to either constant 24-h light (LL) or 12-h light/dark cycles (LD). Mice housed in LL experienced a significant increase in gut barrier permeability that was associated with dysregulated ß-catenin expression and altered expression of tight junction (TJ) proteins. Silencing of ß-catenin resulted in disruption of barrier function in SW480 cells, with ß-catenin appearing to be an upstream regulator of the core circadian components, such as Bmal1, Clock, and Per1/2. In addition, ß-catenin silencing downregulated ZO-1 and occludin TJ proteins with only limited or no changes at their mRNA levels, suggesting post transcriptional regulation. Indeed, silencing of ß-catenin significantly upregulated expression of matrix metallopeptidase (MMP)-2 and MMP-9, and blocking MMP-2/9 activity attenuated epithelial disruption induced by ß-catenin silencing. These results indicate the regulatory role of circadian disruption on gut barrier integrity and the associations between TJ proteins and circadian rhythms, while demonstrating the regulatory role of ß-catenin in this process.

## Introduction

With rising disruption of light–dark cycles in human populations due to night-shift work, excessive use of artificial light, and/or travel across different time zones, there is increasing concern for the effects of circadian rhythm misalignment on susceptibility to disease. Circadian rhythms act as ~ 24 h pacemaker for the human body and its individual cells. The “master clock”, so named for its direct synchronization to light and its influence over all other peripheral clocks throughout the body, is located in the suprachiasmatic nucleus (SCN) of the hypothalamus [1]. Although the circadian rhythm runs autonomously in each cell, they can also be influenced by different environmental cues, such as light and food intake [2]. As a whole, circadian rhythms regulate daily cycles, such as sleep and wake, hunger, body temperature, and hormone secretion to anticipate the body’s daily needs [3]. At a molecular level, clock genes and clock-controlled genes regulate the cell cycle through various cellular functions such as cell replication, apoptosis, and DNA repair.

The core members of the mammalian molecular clock are transcription factors Bmal1 and Clock that bind to the E-box promoter and activate the transcription and translation of Per and Cry genes [4–6]. The proteins of Per and Cry dimerize to form a feedback inhibition that halts the initial transcription of Bmal1 and Clock [7–11]. The molecular clock is also regulated by posttranslational modifications, such as the degradation of Per and Cry proteins via targeting by SCF ubiquitin ligases [12–14]. The degradation of Per and Cry proteins breaks the feedback inhibition and allows Bmal1 and Clock to act as transcription factors thereby continuing the cycle. Other posttranscriptional/posttranslational modifications include sirtuin 1 (Sirt1), nuclear receptors retinoic acid-related orphan receptor alpha (Rora), and reverse erythroblastosis virus alpha (Reverba) [15–18]. The involvement of Sirt1 may link alterations of circadian regulation with inflammatory responses. Indeed, Sirt1 is a ubiquitously expressed deacylase that regulates, among others, acetylation and thus activation status of nuclear factor-κB (NF-κB), a potent stimulator of inflammation [19]. Moreover, Sirt1 activation remains under a partial control of cellular occludin levels [20]. The importance of these reactions stem from the fact that integrity of tissue and cellular barriers are susceptible to altered cellular redox status [21].

When circadian rhythms are disrupted by chemical or environmental interference, processes regulating homeostasis are altered, contributing to the development of various diseases, such as gastrointestinal diseases, sleep disorders, metabolic syndromes, and cancer formation [22]. Mechanistically, circadian rhythm alterations have been linked to the ß-catenin/WNT-signaling pathway. Indeed, circadian rhythm disrupted mice were demonstrated to exhibit both a decrease in expression of Bmal1 and β-catenin [23]. Bmal1, the major regulator of circadian activity, was shown to stimulate the WNT/β-catenin pathway by enhancing the transcription of β-catenin, decreasing the degradation of β-catenin [24], and downregulating Glycogen synthase kinase-3β (GSK-3β) activity [25]. The overexpression of Bmal1 resulted in an increase in the expression of β-catenin, suggesting that the activation of the Wnt pathway may be the mechanism by which Bmal1 promotes cell proliferation [24]. In fact, circadian disruption can accelerate tumor growth through ß-catenin/WNT-signaling pathway [26, 27]. β-catenin expression can be regulated also by other clock genes. For example, the depletion of Per2 was demonstrated to decrease the expression of β-catenin and promote nuclear β-catenin accumulation [23].

The gut barrier is a multilayer system composed of tight junction (TJ) proteins such as zonula occludens (ZO)-1, tricellulin, and occludin that regulate its functions [28]. TJ proteins are responsible for managing the permeability of the gut barrier by providing a selective seal between the neighboring epithelial cell lining [29]. The outer layer acts as a selectively permeable barrier between the gut and the surrounding tissues. The inner layer discriminates against pathogens and regulates immune responses [30]. Several circadian rhythm-related disorders (e.g., chronic and metabolic stress, cardiovascular disease, sleep deprivation, alcohol use, chronic inflammation) show signs of gut barrier leakiness, suggesting that circadian rhythms may be involved in controlling gut barrier permeability [31–34]. Various circadian clock genes and proteins, including Bmal1, Per 1/2/3, and Clock, are expressed in the gastrointestinal tract and have been linked to gut functions, such as digestion, epithelial renewal, and absorption [35–37]. We [38] and others [39] have reported that circadian rhythm disruption alters gut microbiota towards an increase in pro-inflammatory intestinal bacterial abundance, a decrease in anti-inflammatory intestinal bacterial abundances, and impeded intestinal barrier function [17]. In addition, quorum sensing molecules produced by bacteria can disrupt epithelial barrier integrity [40]. At the same time, the intestinal microbiota undergoing diurnal compositional and functional oscillations may also influence host circadian activity [41], indicating that gut microbiota can also signal back to the circadian clock [42]. Circadian rhythms also effectively control local and systemic metabolic processes and inflammation responses; further implicating alterations of gut permeability with alterations of circadian rhythms [17, 43–46].

The exact mechanistic interrelationships between the circadian rhythm and gut barrier integrity are not fully understood. Therefore, the aim of the present study is to examine the link between circadian rhythm disruption and alterations of gut barrier permeability. Our data indicate that loss of barrier function as the result of circadian disruption is mediated, at least in part, by ß-catenin-induced modulation of circadian clock gene and protein expression as well as upregulation of matrix metallopeptidase (MMP) 2/9 leading to a reduction in TJ protein expression.

## Results

### Disruption of circadian rhythms in mice by constant light

**Fig. 1:**
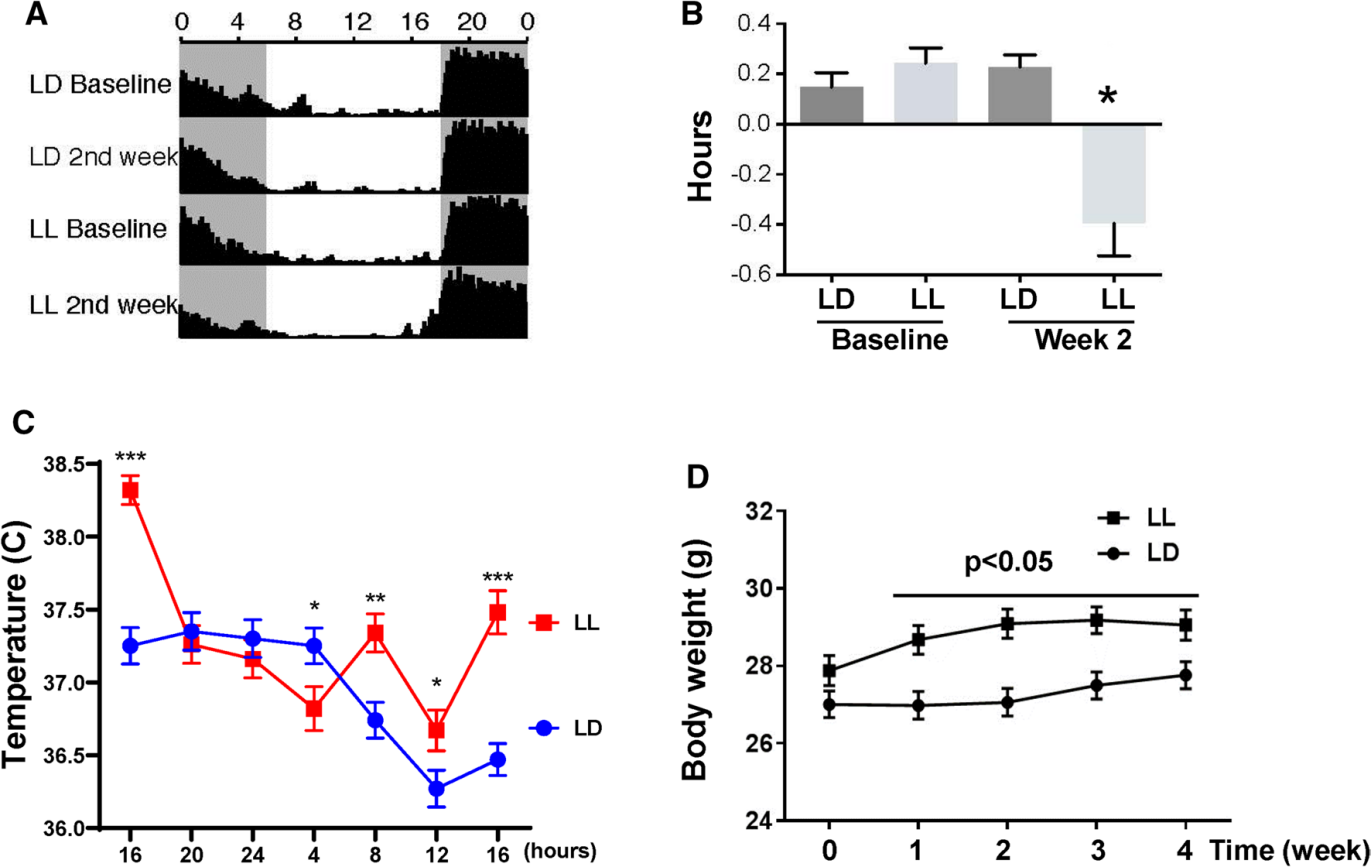
Four weeks of constant light disrupts circadian rhythms in mice. **A** Summary actograms at baseline (before light manipulation) and two weeks after 12-h light/dark (LD) or circadian disruption (constant light, LL). Shaded areas, 18:00 (ZT12) to 6:00 (ZT0), indicate time of lights-off, or former lights-off for LL 2nd week. Numbers along the *x*-axis indicate hours of the day. Black areas are wheel revolutions binned each minute and reported here as the average for *n* = 19 mice per group. **B** Arrhythmicity of exercised mice maintained in LL as demonstrated by phase angle assessment. Phase angle is the difference in hours between the time of lights-off (or former lights-off for LL 2nd week), which was 18:00 (ZT12), and the time of activity onset; corresponding to uninterrupted activity on the wheel. In the LL group at the 2nd week, activity was significantly shifted ~ 30 min before lights-off. **C** Body temperature rhythm measured every 4 h over 24 h in LD and LL mice. **D** An increase in body mass in mice maintained in LL as compared to LD. Values in the LL group that are statistically different from those in the LD group at the corresponding time points at *p < 0.05, **p < 0.01, and **p < 0.001. Values are mean ± SEM

Mice were subjected to constant 24 h light (LL) for 4 weeks, with the control group maintained under 12-h light/dark cycles (LD). Voluntary wheel running was measured to evaluate behavioral circadian rhythms affected by exposure to constant light. All mice showed regular running rhythms at baseline and in normal light (LD) (Fig. 1A). Following two weeks of disrupted light (LL), mice started to display consolidated voluntary running rhythms; however, the time of activity onset was altered (Fig. 1A and B). The phase angle is a circadian parameter that describes the difference in hours between the time of lights-off (or former lights-off for LL 2nd week) which was 18:00 (zeitgeber time [ZT] 12), and the time of activity onset; corresponding to uninterrupted activity on the wheel. In the LL group at the 2nd week, activity was significantly shifted by ~ 30 min (Fig. 1B). These alterations were maintained at constant level throughout the experiment (data not shown).

In addition to wheel running, body temperature was measured as a second method to evaluate circadian rhythmicity. Following two weeks of normal light, mice showed a regular pattern of body temperature, elevated during the night/active time (18:00 [ZT12] until 06:00 [ZT0]) and decreased during the day/resting time. Mice exposed to circadian disruption displayed an arrhythmic pattern in body temperature throughout the day (Fig. 1C).

Body mass was measured at baseline and then once a week for 4 weeks. Mice maintained in normal light exhibited a typical, moderate increase in body weight. On the other hand, mice subjected to circadian disruption showed significantly increased body mass when compared with mice exposed to normal light for 4 weeks (Fig. 1D).

### Circadian rhythm disruption represses the expression of β-catenin and circadian clock genes in the gut

**Fig. 2:**
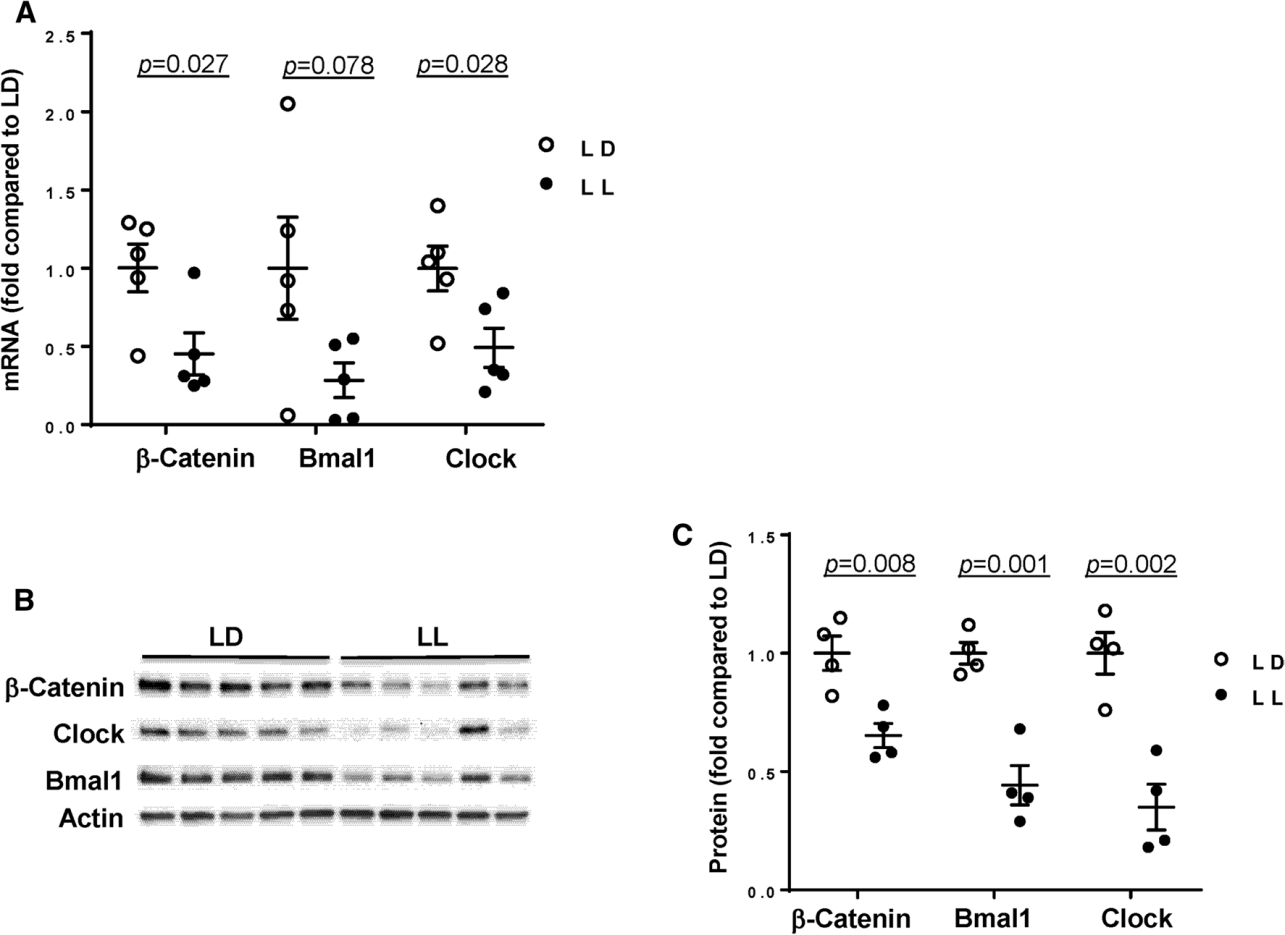
Expression of circadian clock molecules in the gut of the circadian rhythm disrupted mice. The mRNA and protein expression of circadian clock molecules, β-catenin, Bmal1, and Clock were measured in the intestinal epithelial cell-enriched fractions of the colon of mice exposed to 12-h light/dark cycling (LD) or constant 24-h light (LL) for 4 weeks. **A** mRNA expression of β-catenin, Bmal1 and Clock was assessed using RT-PCR. **B**, **C** Protein expression levels from the same mice as (**A**). Protein expression level of β-catenin, Bmal1 and Clock was measured by immunoblotting (**B**) and band intensity was quantified by densitometric analysis using Image J program (**C**). *p* indicates the level of statistical significance in the LL group compared to the LD group for individual genes and proteins. Values are mean ± SEM; *n* = 4–5

We next analyzed the impact of circadian disruption on mRNA (Fig. 2A) and protein expression (Fig. 2B, C) of circadian clock molecules, such as Clock and Bmal1, in control mice and mice with disrupted circadian cycles. These experiments included also β-catenin expression as the WNT/β-catenin pathway was demonstrated to be under a strong circadian control [47] and as described in the Introduction. Alterations of circadian rhythms by LL significantly reduced intestinal Clock and Bmal1 expression at both mRNA levels (by ~ 70% and 50%, respectively) and protein levels (by ~ 55% and 65%, respectively) (Fig. 2A–C). Interestingly, we also detected a significant downregulation of ß-catenin by 65% at the mRNA level and by 35% at the protein levels in the LL group as compared to LD controls.

### Circadian rhythm disruption alters intestinal permeability and TJ protein expression

**Fig. 3:**
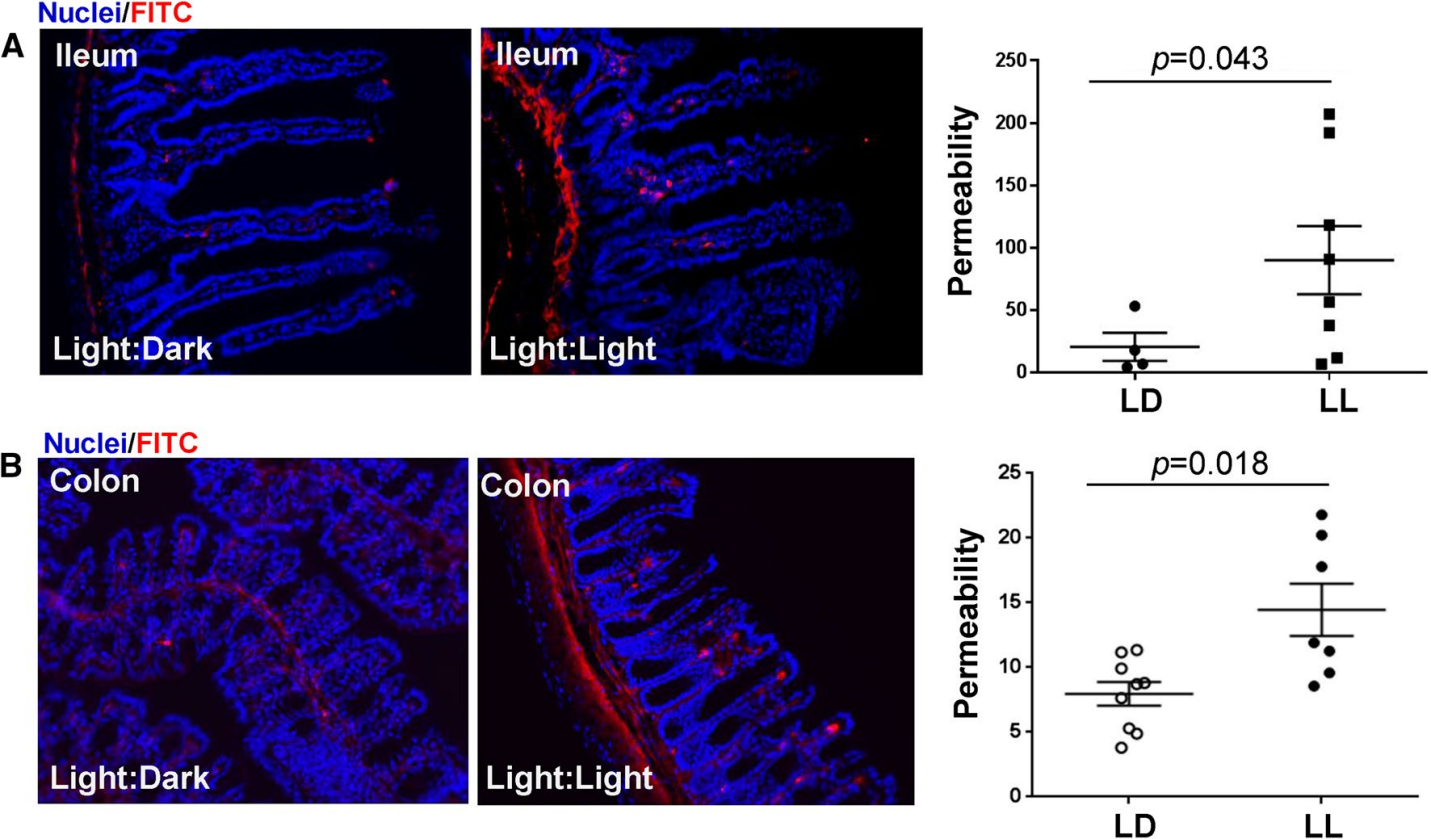
Circadian rhythm disruption alters ileum and colon permeability. Mice were treated as in Figs. 1 and 2. **A** Left panel, representative images of ex vivo permeability to FITC-dextran 4 kDa through the ileum segment in mice maintained under 12-h light/dark (LD) or constant light (LL) for 4 weeks. **B** Ex vivo permeability in the colon in the same mice as in **A**. Left panels, gut sections stained for nuclei (blue) and FITC (red). Right panels, quantitative data from ex vivo permeability measurements. *p* indicates the level of statistical significance in the LL group compared to the LD group. Values are mean ± SEM; *n* = 4–9

Dysfunction of intestinal integrity may be one of the main outcomes of circadian rhythm disruption. Therefore, intestinal permeability was evaluated in the ileum and colon segments in mice maintained under normal and constant light conditions. The analyses were performed ex vivo by perfusion of ileum or colon sections of the intestine with FITC-dextran 4 kDa. The circadian rhythm disrupted group (LL) experienced a significant increase in intestinal permeability as compared to LD controls both in the ileum (Fig. 3A) and the colon (Fig. 3B). Changes in permeability measures were more pronounced and consistent in colon sections; therefore, colon cells were employed in subsequent in vitro experiments in Figs. 5, 6, and 7.

**Fig. 4:**
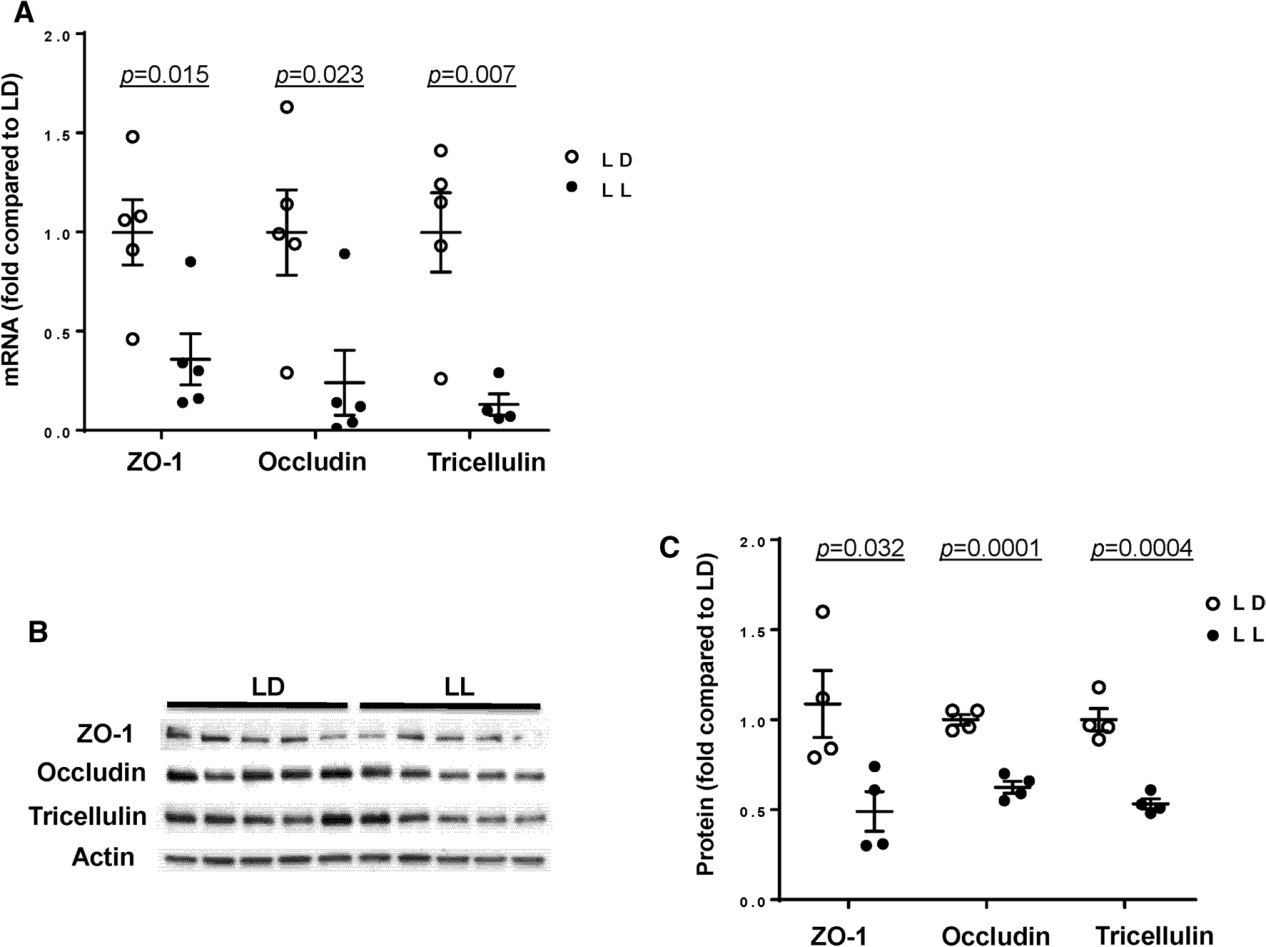
Alterations of tight junction protein expression in the colon of mice subjected to circadian rhythm disruption. The expression of tight junction molecules was measured in the intestinal epithelial cell-enriched fractions of the colon of mice exposed to 12-h light/dark cycling (LD) or constant 24-h light (LL) for 4 weeks. **A** mRNA expression of ZO-1, occludin, and tricellulin as analyzed by real-time PCR. **B**–**D** Protein expression levels from the same mice as (**A**). Protein expression of ZO-1, occludin and tricellulin was assessed by immunoblotting (**B**) and band intensity was quantified by densitometric analysis using Image J program (**C**). *p* indicates the level of statistical significance in the LL group compared to the LD group for individual genes and proteins. Values are mean ± SEM; *n* = 4–5

Alterations in intestinal permeability were accompanied by a significant downregulation of mRNA levels of TJ genes, namely, ZO-1, occludin, and tricellulin (*p* = 0.015, *p* = 0.023, and *p* = 0.007, respectively) (Fig. 4A). The loss of tricellulin is important because tricellulin can normally replace occludin in cell junctions [48]. We next analyzed TJ protein expression by immunoblotting (Fig. 4B, representative blots, Fig. 4C, quantified data). Consistent with the results on mRNA expression, disruption of circadian rhythms markedly downregulated protein levels of all TJ molecules evaluated in the present study by ~ 50% with at the significance level of *p* < 0.032 for ZO-1, *p* < 0.0001 for occludin, and *p* < 0.0004 for tricellulin.

### The expression of the clock molecules is controlled by β-catenin

**Fig. 5:**
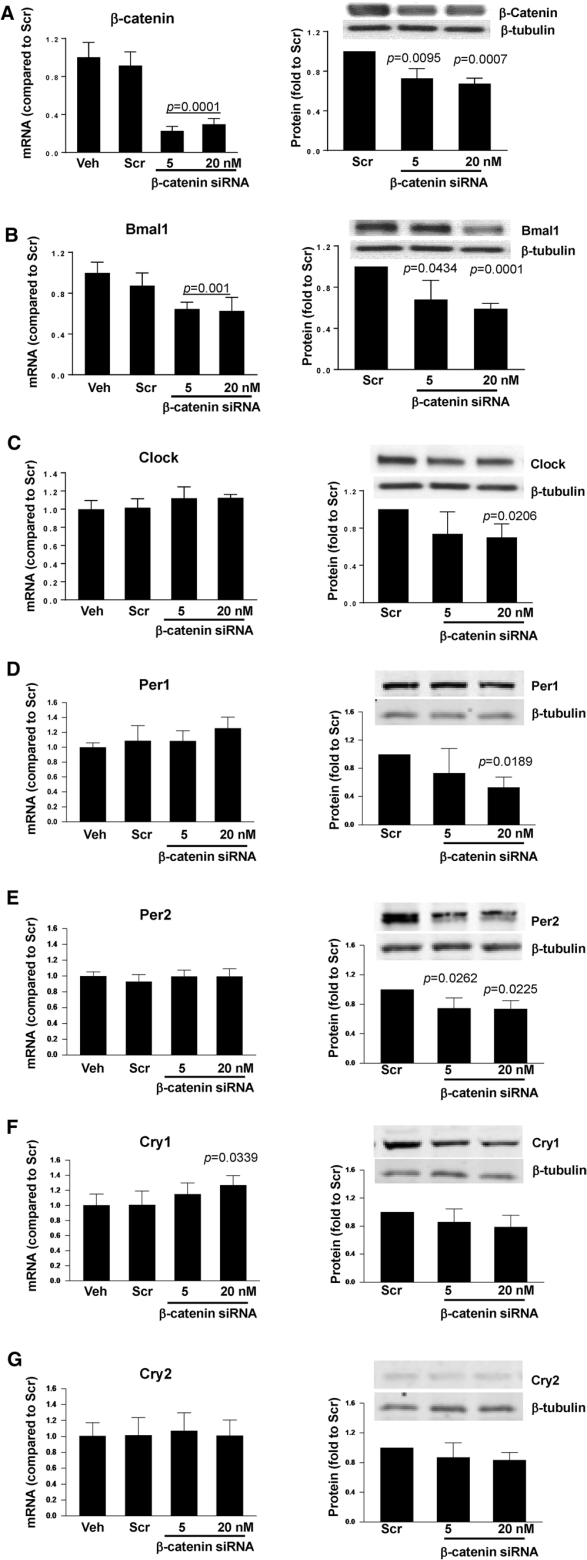
Silencing of β-catenin alters mRNA and protein expression of circadian clock regulators. SW480 cells grown on the 12 well cell culture plates were transfected with β-catenin-specific siRNA at the indicated concentration or with nonspecific, scrambled siRNA (Scr); Veh, vehicle. mRNA (left panels) and protein (right panels) expression of β-catenin (**A**), Bmal1 (**B**), Clock (**C**), Per1 (**D**), Per2 (**E**), Cry1 (**F**), and (Cry2) (**G**) were assayed and quantified. Blots illustrate representative data. Band intensity was assessed by densitometric analysis using Image J program. *p* indicates the level of statistical significance after β-catenin silencing as compared to the Scr group. Values (means ± SD) are expressed as fold change compared with Scr; *n* = 3–6

In order to investigate a potential correlation between β-catenin and clock gene expression, we returned to in vitro studies based on human colon SW480 cells. β-catenin was silenced using specific siRNA, followed by analysis of clock genes mRNA and protein expression (Fig. 5). Silencing β-catenin decreased its mRNA levels by ~ 80% and protein expression by ~ 30% (Fig. 5A, left and right panel, respectively). Interestingly, β-catenin silencing diminished Bmal1 mRNA and protein levels by ~ 40% (Fig. 5B) and Clock protein level by ~ 40% without affecting Clock gene expression (Fig. 5C). Silencing of β-catenin also effectively decreased Per1 and Per2 proteins by ~ 50% and ~ 30%, respectively, without changes in their gene expression levels (Fig. 5D and E). In contrast, Cry1 and Cry2 gene expression and protein levels were unaffected by β-catenin silencing. Overall, these results suggest that β-catenin is upstream from the clock genes and proteins and may modulate their expression in intestinal cells.

### Silencing of circadian clock genes increases epithelial permeability

**Fig. 6:**
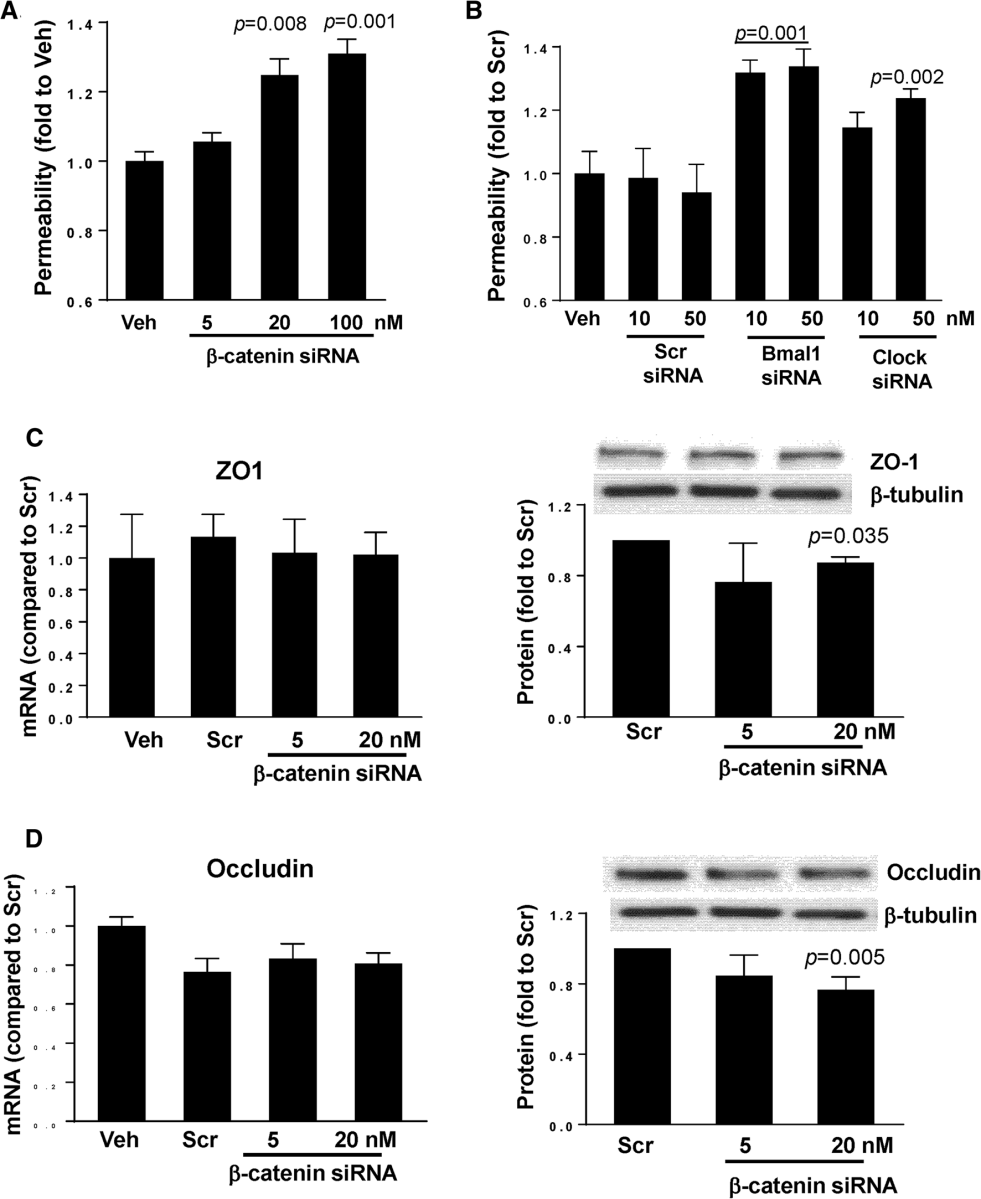
Silencing of circadian clock molecules increases paracellular permeability across epithelial monolayers. SW480 cells grown on the apical side of wells (0.4-μm pore size, 12-mm diameter) of Transwell inserts were transfected with siRNA of β-catenin (**A**), Bmal1, or Clock (**B**) at the indicated concentrations. Control cultures were either treated with vehicle (Veh) or transfected with nonspecific, scrambled (Scr) siRNA. Epithelial permeability was determined by measuring paracellular passage of FITC-dextran 20 kDa from the apical side to the basolateral side across SW480 cell layers. **A** *p* indicates the level of statistical significance after β-catenin silencing as compared to the Veh group. **B** *p* indicates the level of statistical significance after Bmal1 or Clock silencing as compared to the Veh or Scr group. Values (means ± SD) are expressed as fold change compared with Veh or Scr. Values are mean ± SEM; *n* = 5–6. **C**, **D** Silencing of β-catenin alters expression of tight junction proteins without affecting mRNA levels. SW480 cells grown on the 12 well cell culture plates were transfected with β-catenin-specific siRNA at the indicated concentration or with nonspecific, scrambled siRNA. mRNA (left panels) or protein (right panels) expression of ZO-1 (**C**) and occludin (**D**) were assayed and quantified. Blots illustrate representative data. Band intensity was assessed by densitometric analysis using Image J program. *p* indicates the level of statistical significance after β-catenin silencing as compared to the Scr group. Values (means ± SD) are expressed as fold change compared with Scr; *n* = 3–6

To evaluate the impact of clock genes on epithelial barrier function, β-catenin, Clock, or Bmal1 expression was silenced in SW480 cells with specific siRNAs, followed by measuring permeability for FITC-dextran 20 kDa in the Transwell system. Permeability across SW480 monolayers was significantly increased by 24% and 27% upon silencing with ß-catenin siRNA at 20 and 100 nM, respectively (Fig. 6A). In addition, silencing of Bmal1 with specific siRNA at 10 and 50 nM increased permeability by ~ 28%. Silencing the Clock gene with 10 nM siRNA was ineffective, however, silencing with 50 nM significantly increased permeability by 19% (Fig. 6B).

Taking into consideration the substantial impact of β-catenin on the regulation of epithelial barrier function and the role of β-catenin in circadian rhythm regulation, we next analyzed the consequence of β-catenin knockdown on TJ protein expression. Silencing of β-catenin with 20 nM of specific siRNA did not alter ZO-1 mRNA levels; however, it decreased its protein levels (Fig. 6C). Similarly, β-catenin silencing did not affect occludin gene expression. Although there was a ~ 20% reduction in occludin mRNA expression as compared to vehicle, similar downregulation of occludin mRNA was observed in cells transfected with scrambled siRNA, suggesting nonspecific responses. As seen with ZO-1, silencing of β-catenin with 20 nM siRNA significantly decreased occludin protein expression (Fig. 6D).

### β-Catenin regulates epithelial barrier function via upregulation of MMPs

**Fig. 7:**
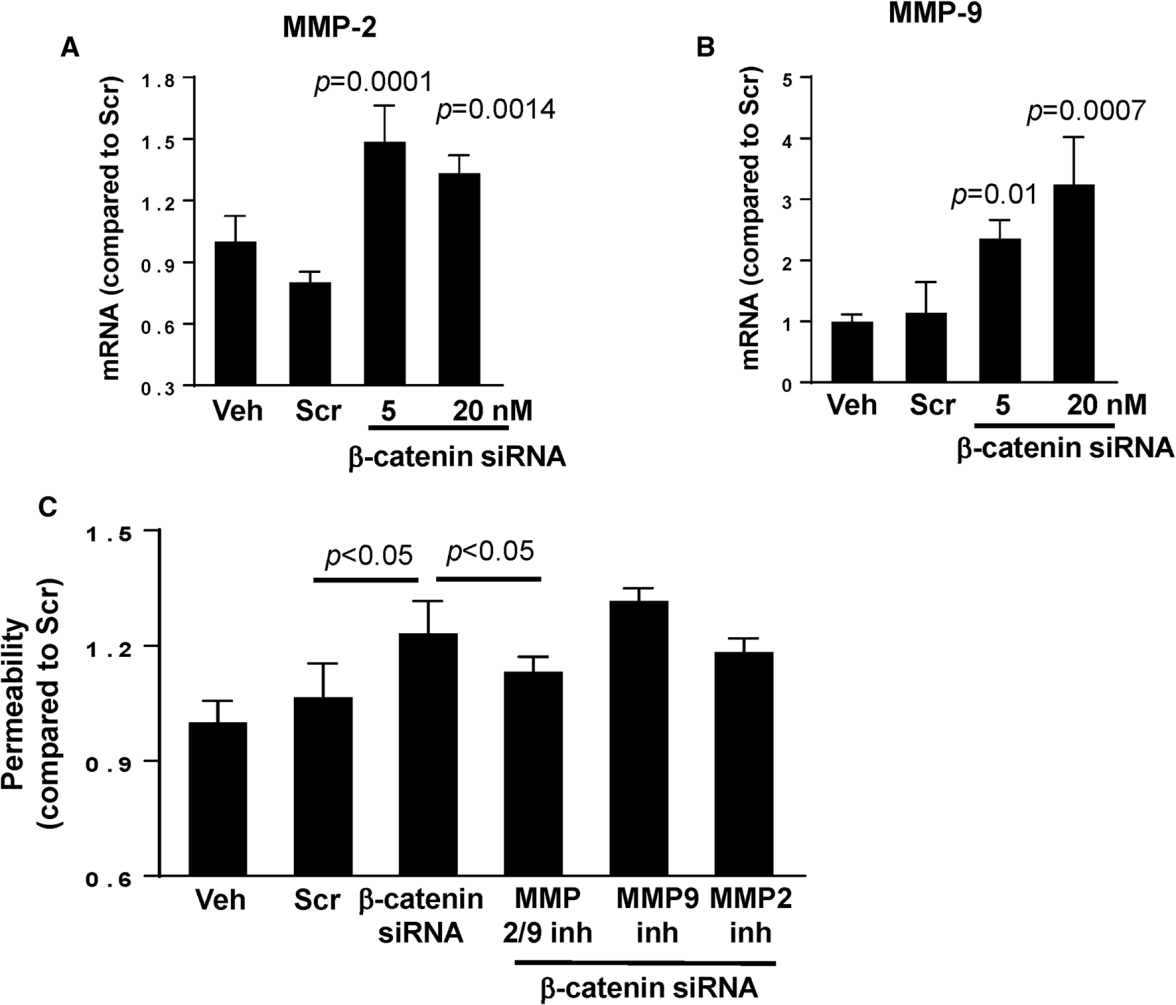
β-catenin-induced alterations of paracellular permeability is mediated by MMPs**.** SW480 cells were transfected with β-catenin-specific siRNA at the indicated concentrations or with nonspecific, scrambled siRNA, followed by mRNA assessment by RT-PCR of MMP-2 (**A**) and MMP-9 (**B**). Values (means ± SD) are expressed as fold change compared with Scr; *n* = 5–6. **A**, **B** *p* indicates the level of statistical significance after β-catenin silencing as compared to the Scr group. **C** SW480 cells grown on the apical side of wells (0.4-μm pore size, 12-mm diameter) of Transwell system were transfected with β-catenin siRNA or scrambled siRNA (both at 20 nM). Additional cultures were treated with pharmacological inhibitors of MMPs, namely, MMP-2/MMP-9 Inhibitor I, MMP-9 Inhibitor II, or MMP-2 inhibitor III all at 20 µM. Epithelial permeability was determined by measuring paracellular passage of FITC-dextran 20 from the apical side to the basolateral side across SW480 cell layers. *p* indicates the level of statistical significance between the β-catenin silencing group as compared to the Scr group or between the β-catenin silencing plus MMP2/9 inhibitor group as compared to the β-catenin silencing group. Values (means ± SD) are expressed as fold change compared to Scr; *n* = 5

A decrease in TJ protein expression without changes on mRNA levels suggests post-transcriptional modification. Therefore, we evaluated a possible involvement of MMPs, which can modulate permeability by degradation of TJ proteins [49]. Transfection with β-catenin siRNA significantly upregulated the mRNA expression of MMP-2 and MMP-9 (*p* < 0.01) (Fig. 7A and B, respectively). In addition, silencing of β-catenin significantly increased permeability across epithelial monolayers created by SW480 cells. In order to determine if increased production of MMPs may be involved in this effect, cells were transfected with β-catenin siRNA and co-treated with MMP-2 or MMP-9 inhibitors. Specific inhibitors for MMP-2 or MMP-9 individually did not affect β-catenin siRNA-induced elevation of epithelial permeability. However, inhibition with a dual-action blocker of both MMP-2 and MMP-9 attenuated disruption of permeability induced by β-catenin siRNA (Fig. 7C), suggesting that MMPs may modulate the impact of β-catenin on epithelial barrier function in the context of circadian disruption.

## Discussion

Circadian rhythms are critically important to maintain normal gut function, to provide intestinal barrier function, to engage bacterial dissemination, and to regulate inflammation, which when disrupted can have several pathophysiological consequences [50–54]. The exact mechanisms linking circadian rhythm disruption and gut barrier integrity are not yet fully understood. Detailed assessment of the gut barrier’s TJ proteins and their interactions with core clock genes provides a better understanding of the relationship between circadian rhythm disruption and gut barrier integrity. Identification of the mechanisms of such interactions is crucial for facilitation of treatment in diseases that are influenced by circadian disruptions.

The gut barrier is vital for maintaining homeostasis and performing functions such as providing a selective barrier, degrading pathogens and antigens, preventing bacterial adhesion and colonization, and initiating an immune response [55, 56]. Circadian rhythms within the gut anticipate these daily needs by signaling to tight junction proteins and other basal membrane proteins in the matrix to aid in intestinal absorption, produce antimicrobial substances, gastric acid, pancreatic juice, and secrete biliary fluids [56, 57]. When circadian rhythms are disrupted, signaling pathways become dysregulated and gut barrier integrity is compromised. Our experimental findings align with observations demonstrating that disruption of normal light cycles in mice results in increased gut barrier permeability (Fig. 3) [58]. It was also observed that LL shifted control-fed mice had similar levels of intestinal hyperpermeability as LD nonshifted alcohol-fed mice [58]. This suggests that the disruption of the circadian rhythm via light cycle shifts has comparable results on gut barrier permeability as chronic alcohol consumption. According to epidemiological data and comparative studies, several gastrointestinal diseases such as chronic inflammatory bowel disease, gastroesophageal reflux disease, irritable bowel syndrome, and peptic ulcers are more common in shift workers with disrupted circadian rhythms [59–62].

In the present study, circadian rhythm disruption through light cycle manipulation led to dysregulation of ß-catenin and circadian rhythm molecules Bmal1 and Clock as well as disrupted TJ protein expression of occludin and ZO-1 at both the mRNA and protein levels (Figs. 2 and 4). These results suggest that the circadian rhythm may regulate gut barrier integrity via a ß-catenin pathway. From a transcriptional standpoint, our findings showed that siRNA-mediated knockdown of β-catenin expression resulted in significant downregulation of Bmal1 at both mRNA and protein levels (Fig. 5B). In contrast, β-catenin silencing affected only protein levels of Clock, Per1, and Per2, without affecting their gene expression. The reasons for this selective downregulation of circadian proteins are not clear; however, they may suggest post-translational degradation mechanisms similar to those involved in downregulation of TJ protein levels (Fig. 6). On the other hand, it is possible that the measurements at a single time point could miss an earlier time frame when gene expression levels could have been affected by β-catenin silencing. Indeed, gene expression changes are frequently more dynamic and temporary as compared to more stable protein expression alterations. Circadian regulation occurs via the circadian autoregulatory loop that controls both protein turnover and subcellular localization of proteins [13].

Interestingly, it was demonstrated that Bmal1 and Clock act as transcription factors that bind directly to the E-box elements of occludin promoters and induce transcription in both mice and human small intestinal epithelial cells [51]. Furthermore, other studies have found that mRNA levels of occludin show time-dependent variation in wild type mice, while a lack of such oscillation accompanied by an increase in gut barrier permeability has been observed in circadian rhythm disrupted mice (Clock^Δ19/Δ19^) [51, 58].

Circadian regulation of other barriers, such as in the inner blood–retina barrier (iBRB), has also been demonstrated. For example, TJ protein claudin-5 was found to be under control of the core clock in studies in which RNAi-mediated knockdown of Bmal1 affected the integrity of the iBRB [63]. Additional observations indicated that mice were more resistant to *Salmonella* infection and disruption of gut barrier permeability when ß-catenin expression was modified to be constitutively active in intestinal endothelial cells, altering occludin and ZO-1 expression [64]. The findings of the present study are in line with these results indicating that ß-catenin has a regulatory effect on gut barrier integrity. Indeed, silencing ß-catenin resulted in an increase in gut barrier permeability (Fig. 6A). Permeability of the gut barrier also increased when Bmal1 and Clock were silenced, further demonstrating a link between circadian rhythm and gut barrier integrity (Fig. 6B). However, only protein levels of ZO-1 and occludin (Fig. 6C and D) were found to significantly decreased with ß-catenin knockdown, suggesting that expression of these TJ proteins is regulated via post-translational mechanisms.

We further investigated which post-translational mechanisms may be involved and found that ß-catenin knockdown led to higher mRNA expression levels of MMP-2 and MMP-9 as well as an increase in gut barrier permeability (Fig. 7). MMPs are endopeptidases that regulate extracellular matrix homeostasis [65]. MMP-2/9 specifically are gelatinases that activate pro-inflammatory agents and mediate TJ protein degradation [66, 67]. Our observations are in agreement with the involvement of MMPs in the regulation of tissue barrier integrity as shown previously by us [68] and others [69]. ß-catenin has been linked to MMP-2/9 in epithelial cells via negative regulation as inhibition of Wnt/ß-catenin signaling led to increases in extracellular matrix metalloproteinase inducer (EMMPRIN) and MMP-2/9. In addition, activation of Wnt/ß-catenin signaling resulted in large decreases in EMMPRIN and MMP-2/9 [70]. Although ß-catenin appears to control the integrity of epithelial monolayers via MMP2/9 activity, alternative molecular pathways have also been suggested to maintain gut barrier integrity, such as sonic hedgehog (SHH) signaling and endothelial to mesenchymal transition (EndoMT) signaling [64].

## Conclusion

**Fig. 8:**
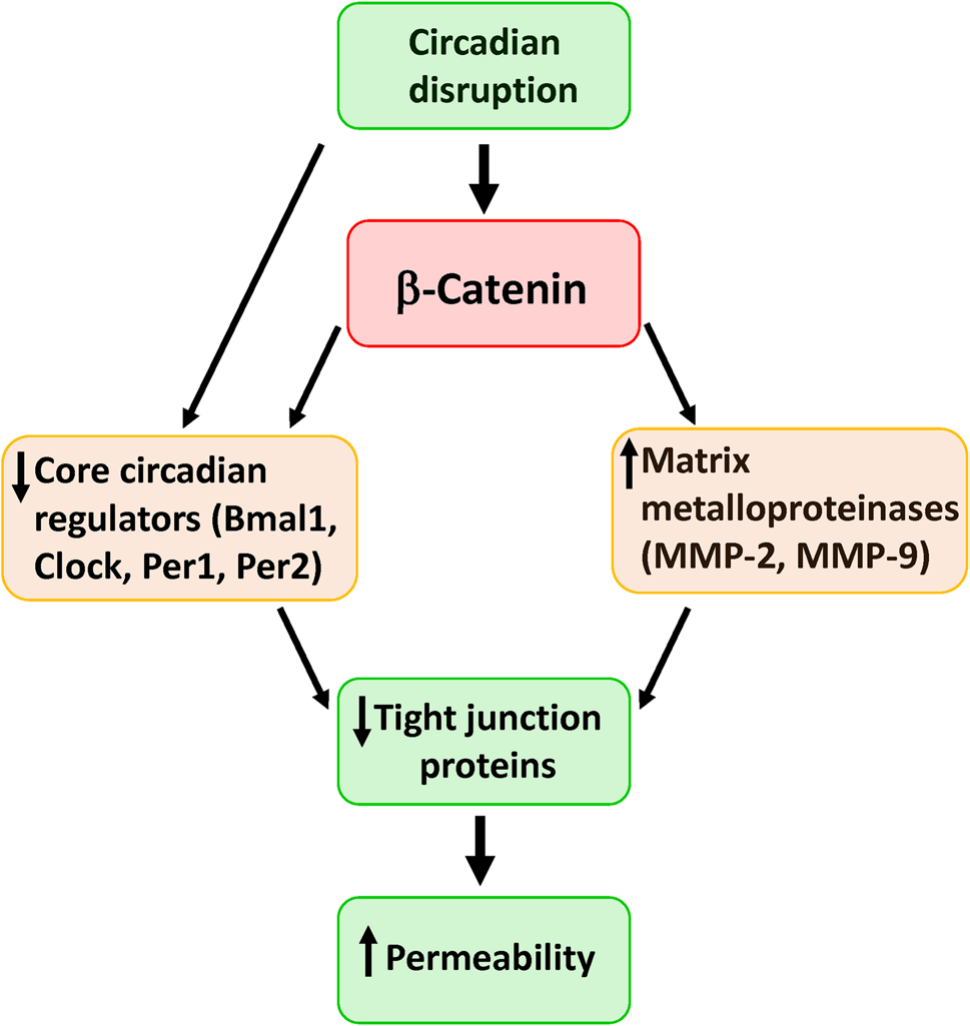
Schematic diagram of the major findings of the present manuscript. ß-catenin appears to be a master regulator of epithelial barrier integrity in circadian rhythm disruption by altering TJ protein expression

Circadian rhythm disruption induced by light cycle manipulation resulted in decreased gut barrier integrity concurrent with dysregulation of TJ proteins. Silencing of ß-catenin and core clock genes, such as Bmal1 and Clock increased gut barrier permeability, implicating essential clock involvement in gut barrier regulation. Interestingly, ß-catenin appears to be a master regulator of this process by acting upstream from Bmal1 and Clock and altering expression of MMP2/9 (Fig. 8).

## Methods

### Animal housing

Male C57BL/6 mice (Jackson Labs, Bar Harbor, ME), 12 weeks of age, were allowed to acclimatize to the animal facility for four weeks with free access to food and water. Mice were randomly assigned to normal lighting (light/dark, LD) and constant lighting (light/light, LL) conditions for four weeks. Throughout the study, all mice had unlimited access to food and water. Mice were euthanized with carbon dioxide followed by decapitation and collection of small and large intestine.

### Ethics statement

All animal procedures were approved by the University of Miami Institutional Animal Care and Use Committee and performed in accordance with National Institutes of Health (NIH) and the American Association for Accreditation of Animal Care (AAALAC) guidelines and regulations. Moreover, the study was carried out in compliance with the Animal Research: Reporting of In Vivo Experiments (ARRIVE) guidelines.

### Circadian manipulation and circadian rhythm measurements

The circadian disruption protocol, constant light for 4 weeks, was intended to disrupt normal circadian rhythms in a similar manner that could be observed in persons in intensive care units living under constant illumination. The objective was to create an environment with no light entraining cues (disruption) when compared with normal day/night light cycling experienced by the control mice in 12-h light/12-h dark. The illuminance was measured in and around the cages where animals were being housed. The same lighting source (fluorescent lights on ceiling) was used in both LD and LL rooms. Light was measured using a light meter (VWR International, Radnor, PA) in and around the cages ranged from 400 to 600 lx. The lighting schedule for LD mice was 12 h of light followed by 12 h of darkness, lights on at 6AM (ZT0) and lights off at 6PM (ZT12), Eastern Standard Time.

Mice were initially housed under normal cycling light (LD) as described above. For four days they were allowed to acclimate to single housing, diet, and environment, then baseline circadian rhythms were measured by collecting rectal body temperature every 4 h for 24 h. Following collection of body temperature, mice were singly housed in running wheels for 48 h to monitor locomotor behavioral rhythms. After 48 h mice were randomized to LD or LL. Mice were maintained in these rooms for two weeks at the end of which, circadian rhythms were measured a second time by collecting rectal body temperature using a rectal thermometer (Acorn series, OAKTON Instruments, Vernon Hills, IL). Body temperature was measured at the same time points for both groups, 16:00 (ZT10), 20:00 (ZT14), 24:00 (ZT18), 04:00 (ZT22), 08:00 (ZT2), and 12:00 (ZT6), Eastern Standard Time.

Voluntary running was measured twice during the experiment (at baseline and after 2 weeks of circadian disruption). Activity rhythms were monitored using plastic cages measuring 30.5 × 15.2 × 12.7 cm containing a running wheel (Coulbourn Instruments, Whitehall, PA). During the time in wheel cages mice had voluntary access to the running wheel as well as food and water. Wheel revolutions were counted on an attached computer using Clocklab software (Actimetrics, Wilmette, IL). Mice had no prior exposure to the running wheels before the experiment began, and running was voluntary.

### Ex vivo and in vitro intestinal permeability

Intestinal permeability was measured ex vivo in isolated ileum and colon segments. Briefly, 6-cm segments of the ileum and the colon were removed, rinsed with ice-cold PBS, filled with 700 μl DPBS containing 2 mg/ml FITC-dextran 4 kDa and ligated at both ends. The filled intestine segments were incubated in DMEM containing 1% FBS. The sacs were removed after 45 min. The amount of FITC-dextran that transversed the intestine was quantified by fluorescence plate reader at Ex 485 nm and Em 530 nm. In addition, ileum and colon segments were sectioned and stained for nuclei (DAPI). Fluorescent images visualizing FITC-dextran 4 kDa and nuclei were taken using confocal microscopy.

SW480 cells were seeded on collagen type I-coated Transwell polyester filters in 5 × 10^5^ density (12-mm diameter, 0.4 µm pore size, Corning Costar), transfected with β-catenin, Bmal1, Clock, or scrambled (scr) siRNA. In selected experiments, MMP inhibitors were added to both the lower and the upper compartments of the Transwell system. Then, 0.5 ml of FITC-dextran 20 kDa (FD-20, 1 mg/ml in KRG solution) was loaded into the upper chamber, the system was allowed to incubate for 60 min at 37 °C in humidified atmosphere (5% CO_2_), and the assay was stopped by removing the upper chambers. Aliquots (100 μl) from the lower chambers were transferred to new wells of 96-well fluorescence plate, and the fluorescence intensity of FITC-dextran was determined with a microplate spectrofluorometer (Molecular Devices SPECTRA-Max Gemini EM) using 490 nm and 520 nm as excitation and emission wavelengths, respectively. Relative permeability was expressed by the ratio of FD-20 transported into the lower chamber as compared to control groups. All assays were performed at least in quadruplicate.

### Cell culture, treatment, and transfections with small interfering RNA

SW480, epithelial colonic cancer cells, were purchased from the American Type Culture Collection (ATCC, Manassas, VA) and cultured in ATCC-formulated Leibovitz's L-15 Medium (Catalog #30-2008). In selective experiments, cells were pretreated for 0.5–1 h with pharmacological inhibitors of MMPs, including MMP-2/MMP-9 Inhibitor I, MMP-9 Inhibitor II, MMP-2 inhibitor III (Millipore Sigma) at 20 µM.

SW480 cells were transfected with control siRNA or targeted siRNA at indicated concentrations using Lipofectamine 2000 (ThermoFisher) in OptiMEM I medium (Invitrogen, Carlsbad, CA). Cells were incubated with transfection mixtures for 6–20 h and allowed to recover in complete medium for 48 h before the assays.

### Real-time RT-PCR

The total RNA was isolated and purified using RNeasy Mini Kit (Qiagen) according to the protocol of the manufacturer. Then, 1 µg of total RNA was reverse transcribed at 25 °C for 15 min, 42 °C for 45 min and 99 °C for 5 min in 20 µl of 5 mM MgCl_2_, 10 mM Tris–HCl, pH 9.0, 50 mM KCl, 0.1% Triton X-100, 1 mM dNTP, 1 unit/µl of recombinant RNasin ribonuclease inhibitor, 15 units/µg of AMV reverse transcriptase, and 0.5 µg of random hexamers. For quantitative PCR, amplifications of individual genes were performed on ABI PRISM® 7000 Sequence Detection System (Applied Biosystems, Foster City, CA) using TaqMan® Universal PCR Master Mix, gene-specific TaqMan PCR probes and primers, and a standard thermal cycler protocol (50 °C for 2 min before the first cycle, 95 °C for 15 s and 60 °C for 1 min, repeated 45 times). The primers and probes were obtained from Applied Biosystems. The threshold cycle (*C*_T_) from each well was determined using ABI Prism 7000 SDS software. Relative quantification, which represents the change in gene expression from real-time quantitative PCR experiments between treated and control groups, was calculated by the comparative C_T_ method as described earlier [71]. The data were analyzed using equation ${2}^{{{-}\Delta \Delta C_{{\text{T}}} }}$, where ΔΔ*C*_T_ = [*C*_T_ of target gene − *C*_T_ of housekeeping gene]_treated group_ − [*C*_T_ of target gene − *C*_T_ of housekeeping gene]_untreated control group_. For the treated samples, evaluation of ${2}^{{{-}\Delta \Delta C_{{\text{T}}} }}$ represents the fold change in gene expression, normalized to a housekeeping gene (β-actin) and relative to the untreated control.

### Immunoblotting

Protein expression levels of β-catenin, Bmal1 and Clock, Per1 and Per2, Cry1 and Cry2, ZO-1, occludin and tricellulin were assessed in the intestinal epithelial cell-enriched fractions of the isolated colon or in SW480 cells by immunoblotting. All primary antibodies were purchased from ThermoFisher, and HRP-conjugated secondary antibodies were obtained from Santa Cruz Biotechnology (Santa Cruz, CA). Briefly, gut homogenates or treated cells were lysed with RIPA lysis buffer (1.0% Nonidet P-40, 0.5% deoxycholic acid, 0.2% SDS, 40 mM Tris–HCl [pH 7.6], 1 mM EDTA, 1 mM EGTA, 10 mM MgCl_2_, 150 mM NaCl, 1 mM Na_3_VO_4_, 1 mM NaF, 1 × EDTA-free protease inhibitor cocktail [Roche Applied Science], and 1 mM phenylmethylsulfonyl fluoride) for the total cell extract. Protein concentration was determined using BCA protein assay kit (Thermo Scientific, Rockford, IL). Then, 10 μg protein of cell lysates was electrophoresed on SDS–polyacrylamide gels, transferred to a polyvinylidene fluoride (PVDF) membrane, blocked with 3% BSA in PBS-T (0.1% Tween-20) solution and incubated with the primary antibodies overnight at 4 °C. After incubation with the secondary antibody for 2 h, immunoblots were visualized using the ECL detection system (Amersham Biosciences). GAPDH or actin was determined as the loading control. The band density was measured using Image J software (NIH).

### Statistical analysis

The data were statistically analyzed using one-way ANOVA, followed by Tukey’s multiple comparisons test. Statistical probability of *p* < 0.05 was considered statistically significant. The results are expressed as means ± S.D. All experiments were repeated at least 3 times.

## Data Availability

All source data supporting the findings of this manuscript are available from the corresponding author upon request.
